# A Case Report and Review of the Literature: Reactive Arthritis Caused by *Clostridioides difficile ribotype 027*

**DOI:** 10.3389/fmicb.2022.837422

**Published:** 2022-02-16

**Authors:** Ortrud Zimmermann, Heinrich Köchel, Wolfgang Bohne, Beatrix Pollok-Kopp, Peter Passenberg, Uwe Groß

**Affiliations:** ^1^Institute for Medical Microbiology and Virology, University Medical Center Göttingen, Göttingen, Germany; ^2^Department of Transfusion Medicine, University Medical Center Göttingen, Göttingen, Germany; ^3^St. Martini Hospital, Clinic for Internal Medicine and Gastroenterology, Duderstadt, Germany

**Keywords:** *Clostridioides difficile*, *Clostridium difficile*, CDI, reactive arthritis, CDARA, IgA antibodies

## Abstract

With an annual incidence of 250-300 per 100,000 inhabitants, reactive arthritis is not uncommon. However, the fact that *Clostridioides difficile* infection (CDI) can also lead to this complication is largely unknown. We report on a 69-years-old man who developed reactive arthritis of his right knee joint one week after antibiotic-associated diarrhea with evidence of *C. difficile* of the hypervirulent ribotype 027. His female partner also became infected with *C. difficile* ribotype 027, but did not develop reactive arthritis. The further investigation showed that the patient - in contrast to his partner - was HLA-B27 positive and had strong antibody levels against *C. difficile*. The case history together with the review of 45 other cases described so far shows that *C. difficile* can also lead to reactive arthritis. *C. difficile*-associated reactive arthritis (CDARA) is characterized by the fact that patients suffer from diarrhea or colitis after taking antibiotics, toxigenic *C. difficile* or only the toxins are detectable in the stool and there are no other explanations for the arthritis and diarrhea.

## Introduction

The frequency of *Clostridioides difficile* infections (CDIs) has been increasing worldwide for more than 15 years (Rupnik et al., 2009). It is assumed that there are at least 1,500 severe CDIs in Germany per year, with regional incidences varying between 0.2 (State of Saarland) and 6.6 diseases/100,000 inhabitants (State of Saxony-Anhalt). The most accepted typing scheme of *C. difficile* is based on PCR ribotyping. Like has been described for the general prevalence of *C. difficile*, large variation of 0.6 to 37.4% has also been found in Germany for the prevalence of ribotype 027 (Marujo and Arvand, 2020), with a 10.3% prevalence rate of this *C. difficile* ribotype in the State of Lower Saxony (Seugendo et al., 2018). 418 (26%) of the patients registered in Germany in 2020 did not survive their illness (Robert-Koch-Institute [RKI], 2020). In the United States, around 30,000 deaths per year have been reported (Lessa et al., 2015). Almost all clinically manifest CDIs develop colitis, the symptoms of which range from simple inflammation of the mucosa to a pseudomembranous form or even a megacolon (Lübbert et al., 2014). Extraintestinal forms such as, for example, reactive arthritis have hitherto been viewed as a very rare complication. Therefore, *C. difficile* is mostly overlooked as the cause of this potential secondary disease (Birnbaum et al., 2008; Mattila et al., 2013). Up to date - together with our patient - a total of 46 cases of *C. difficile*-associated reactive arthritis (CDARA) have been described. However, information on the respective ribotype that has caused this complication is only given for one other case, making the contribution of ribotype 027 for the development of CDARA unclear at the moment.

## Case Presentation

A 69-years-old man from the State of Lower Saxony, Germany, presented at his doctors practice because of mushy stool and pain in the lower abdomen that had persisted for several days. At this time, no diagnosis of *C. difficile* infection was done. However, abdominal sonography showed clear signs of sigmoid diverticulitis with a considerable pericolic inflammatory reaction, whereupon the patient was treated intravenously with piperacillin/combactam (3 g × 4.5 g daily) for five days. The diagnosis could be confirmed by computer tomography. His inflammation parameters decreased significantly during antibiotic therapy (drop in CRP from 100 mg/l to 20 mg/l). The antibiotic therapy had to be administered orally for the upcoming discharge. Therefore, the patient was prescribed ciprofloxacin 250 mg (1-0-1) for an additional seven days.

Under this therapy, the patient developed intestinal symptoms as early as the third day after discharge with watery, not bloody, but painful diarrhea, which occurred up to twenty times a day during the worst phase. Stool samples were cultivated on *C. difficile* selective agar (bioMérieux, Marcy-l’Étoile, France) for 48 hrs under anaerobic conditions. Suspected colonies were sub-cultured on COS Columbia blood agar that was enriched with 5% sheep blood (bioMérieux). *C. difficile* was identified with score values ≥ 2,000 using MALDI-TOF mass spectrometry (Biotyper, Bruker Daltonics, Bremen, Germany). Bacterial toxins were determined using the Quik Chek Complete (Alere Techlab, Blacksburg, United States). PCR ribotyping was performed by Maja Rupnik, Slovenia, by capillary gel electrophoresis as described before (Seugendo et al., 2018). These examinations showed as cause of this diarrhea both the cultural proof of *C. difficile* ribotype 027 and the presence of toxins A and B. No other intestinal pathogens were detected. Since oral metronidazole therapy quickly improved the intestinal symptoms, the patient and his partner, who lived in the same household, decided to go on vacation to an island in the Northern Sea a week later. The drive there went without any further health problems; even immediately before the crossing to the island, no joint problems were yet evident.

Immediately after arrival at their holiday home, however, his right knee was so painfully swollen that he could hardly move it and would have liked to break off the vacation he had just started. In the course of the 14 days holidays, the symptoms of diarrhea improved after taking a bland diet and a preparation containing *Saccharomyces boulardii* (Perenterol ^®^). Therefore, he decided not to break off his vacation and not to see a doctor on the island, although the knee joint was still painfully swollen and restricted in movement. According to the patient’s memory, he had already suffered reactive arthritis of the right knee joint after an enteric *Salmonella* infection more than 30 years ago. After his return from vacation, a previously planned total colonoscopy was performed, but the result was normal. Just five days after the colonoscopy, the patient developed another flare-up of severe intestinal infection with a high fever and severe diarrhea, so he sought medical advice.

Physical findings: We saw a 168 cm tall, 66 kg heavy patient in a reduced general condition. He stated that he had lost 9 kg in body weight and suffered from pain in the right knee joint within the last six weeks due to febrile diarrhea episodes. The function of the right knee was slightly terminally impaired in a side comparison. The cardiopulmonary auscultation findings and ECG were normal.

**FIGURE 1:**
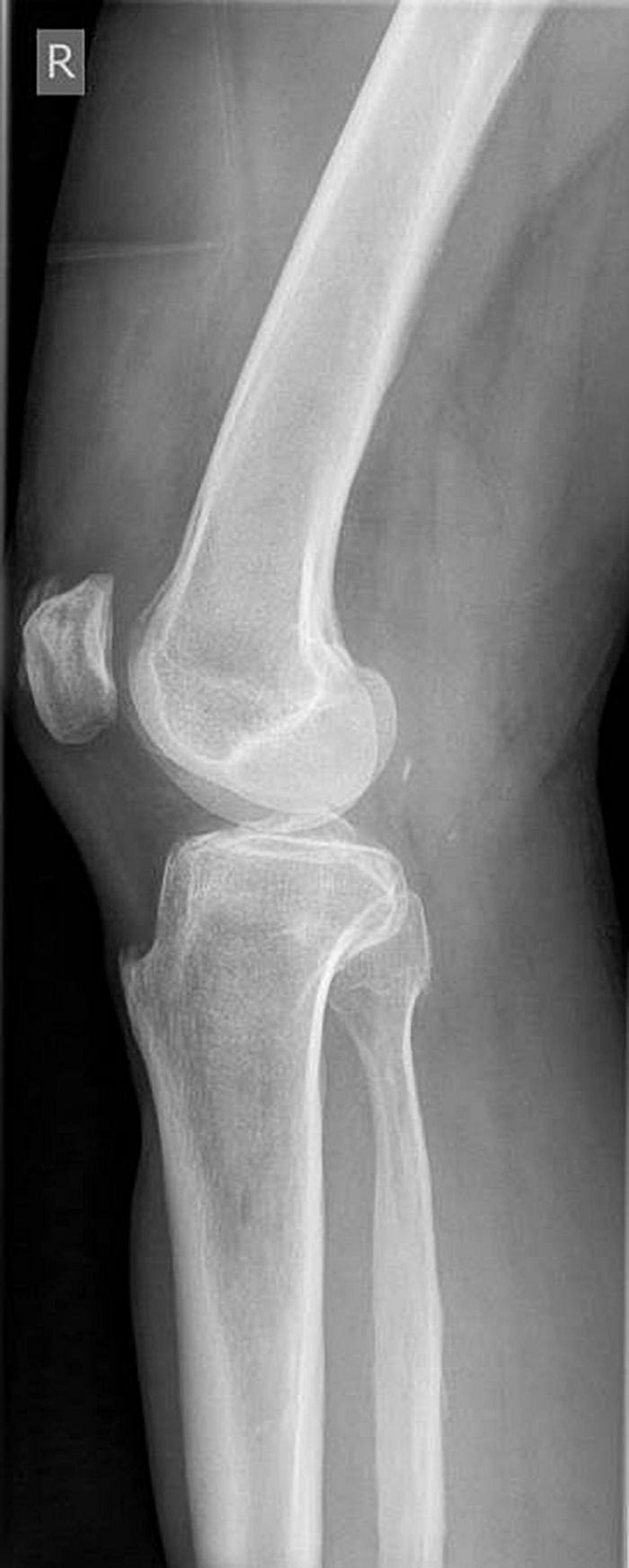
X ray of the right knee.

Diagnostic findings: Due to the pain in the right knee joint described in the anamnestic, a duplex sonography of the deep leg veins on the right was performed, which could rule out a thrombosis, but showed a slight knee joint effusion on the right. The microbiological examination of the stool again revealed *C. difficile* ribotype 027, so that it was most likely a relapse of its original CDI. The X-ray findings of his right knee joint still showed a slight swelling of the capsule, but no relevant joint effusion. The function was ultimately slightly impaired in a comparison of the sides (Figure 1).

Intervention: With oral vancomycin therapy, his symptoms finally improved within a few days. The knee pain was treated conservatively with diclofenac resinate and local compresses with Kytta plasma for the night.

Follow-up and outcome: Further examinations did not reveal any evidence of persistent CDI in the patient. The 63-years-old female partner, who lives in the same household, was treated orally with ampicillin for a tooth abscess at the time of our patient’s relapse. As a result, five days after the start of ampicillin therapy, she developed watery, non-bloody and hardly painful diarrhea, which was treated orally with metronidazole one day after the onset of symptoms. However, since the symptoms initially improved only slightly, she also provided a stool sample for microbiological analysis. The test for *C. difficile* toxin A/B was positive; the cultural examination confirmed also a CDI with a RT027 strain. Since (i) *C. difficile* ribotype 027 is relatively rare in the State of Lower Saxony, (ii) both patients live as a pair in the very same household, and (iii) both were infected by an identical ribotype 027 at the very same time, it is very likely that they were infected by the same strain. However, since we did not perform whole genome sequencing at that time, final proof for strain identity could not be achieved. In contrast to her partner, this female patient suffered only from moderately severe diarrhea, which, however, dragged on for two months and was not accompanied by any joint involvement. Metronidazole was only taken intermittently for seven days due to poor tolerability. In addition, she did not consult a doctor because she did not suffer from fever or significant weight loss.

**FIGURE 2:**
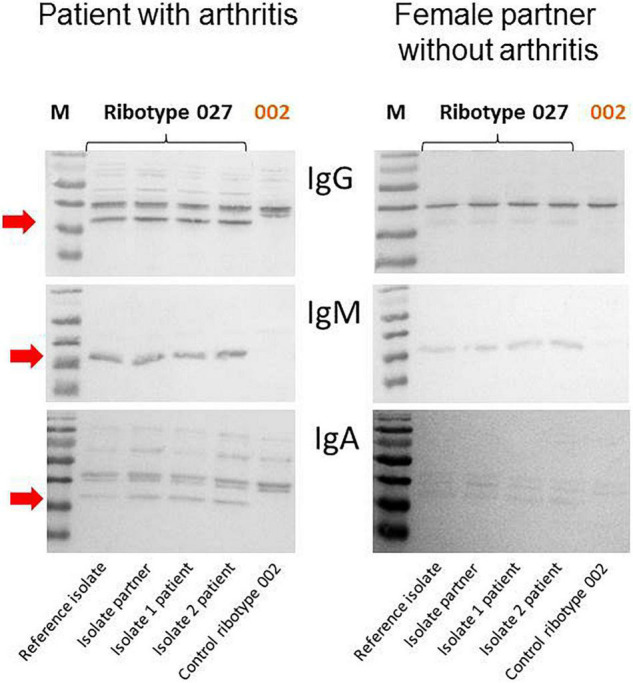
Strong humoral immune response of the patient against SlpA of three RT027 strains (reference strain, isolate of the female partner, two isolates of the patient) but not against a ribotype 002 control isolate. The arrow marks the humoral immune response against the immunodominant protein SlpA.

Analytics: Due to the diarrhea symptoms of the patient and his partner and the detection of toxigenic *C. difficile* strains in the examined stool, the bacterial isolates were ribotyped using PCR. Since the identical hypervirulent ribotype 027 of clade 2 was involved in both cases, it is very likely that the partner living in the same household had been infected by the patient. Interestingly, only the male patient developed reactive arthritis, and his intestinal symptoms were much more severe than those of his female partner. For this reason, we assumed an immunopathological reaction as the cause of both the more severe intestinal symptoms and the development of reactive arthritis and examined the humoral immune response of the patient and his partner to *C. difficile* in an immunoblot (Pantosti et al., 1989). It was shown that the patient - in contrast to his partner - had a strong humoral immune response to the low-molecular-weight S-layer protein SlpA from *C. difficile*, which is considered to be immunodominant (Figure 2).

## Review of the Literature and Discussion

We performed a literature search on PubMed Central to identify articles published as of December 14, 2021. With the help of the key words “*Clostridium difficile*, *Clostridioides difficile*” in combination with “reactive arthritis”, 53 articles could be identified. Of these, 19 were removed because they were reviews, focused on basic science studies, the patients described had co-infections or no reactive arthritis, leaving 34 relevant articles for discussion (Table 1). In addition, articles were included that are important in understanding the possible pathogenesis of *C. difficile*-associated reactive arthritis (CDARA).

**TABLE 1:** Published cases of *C. difficile*-associated reactive arthritis (CDARA) in chronological order.

No	Age	Sex	Affected joint(s)	Pre-existing antibiosis	Reason for antibiosis	HLA-B27	Results from stool analysis	Year	References
1	69	m	Knee	piperacillin/ combactam, ciprofloxacin	sigmadiverticulitis	+	culture + toxin AB + ribotype 027	2021	this case
2	20	m	Polyarthralgia	none	none (psoriasis)	+	toxin +	2021	Reinholz et al., 2021
3	58	f	Knee	cefuroxim	infection of the upper respiratory tract	+	toxin B +	2018	Marwat et al., 2018
4	40	f	Polyarthralgia	amoxicillin/ clavulanic acid	tooth abscess	n.d.	culture + toxin B +	2017	Garrigues et al., 2017
5	47	m	Hip	amoxicillin/ clavulanic acid	tooth extraktion	n.d.	culture + toxin B +	2017	Garrigues et al., 2017
6	6	m	Polyarthralgia	amoxicillin/ clavulanic acid	infection of the upper respiratory tract	-	culture + toxin A/B +	2016	Cappella et al., 2016
7	61	m	Knee	none	none (hernia)	+	GDH +	2016	Essenmacher et al., 2016
8	46	f	Oligoarthralgia	cefixim	urinary tract infection	n.d.	GDH + culture + toxin B + ribotype 014/020/077	2016	Legendre et al., 2016
9	7	m	Polyarthralgia	amoxicillin/ clavulanic acid	watery diarrhea	-	toxin A/B +	2012	Dacheux et al., 2012
10	43	f	Polyarthralgia	none	none	-	culture - toxin A +	2010	Ben Abdelghani et al., 2010
11	64	f	Knee	amoxicillin/ clavulanic acid	pneumonia	+	toxin +	2010	Prati et al., 2010
12	10	f	Hip	erythromycin, penicillin	tonsillitis	n.d.	toxin A/B +	2009	Durand and Miller, 2009
13	72	m	Oligoarthralgia	levofloxacin, clindamycin	cough	+	toxin +	2008	Birnbaum et al., 2008
14	45	m	Knee	clindamycin, piperacillin/ tazobactam	sigmadiverticulitis with intestinal abscess	-	culture + toxin A +	2005	Ducroix-Roubertou et al., 2005
15	61	f	Ankle	cefuroxim	bronchitis	+	culture + toxin A/B +	2005	Guillemin and Gerster, 2005
16	11	m	polyarthralgia	lincomycin	perioperative prophylaxis	+	culture + toxin AB -	2004	Löffler et al., 2004
17	6	f	Polyarthralgia	none	none	-	culture + toxin A/B +	2004	Löffler et al., 2004
18	22	f	Oligoarthralgia	amoxicillin	rhinopharyngitis	+	toxin +	2000	Vermeulen et al., 2000
19	67	m	Polyarthralgia	lincomycin	bronchitis	+	toxin +	2000	Vermeulen et al., 2000
20	20	m	?	penicillin, ciprofloxacin	?	-	toxin A +	1998	Kocar et al., 1998
21	25	f	?	cephadroxil	?	+	toxin A +	1998	Kocar et al., 1998
22	25	f	?	none	?	n.d.	toxin A +	1998	Kocar et al., 1998
23	23	m	?	ampicillin	?	+	toxin A +	1998	Kocar et al., 1998
24	20	m	?	ampicillin/ sulbactam	?	-	toxin A +	1998	Kocar et al., 1998
25	21	m	?	cefuroxim	?	-	toxin A +	1998	Kocar et al., 1998
26	57	m	Oligoarthralgia	cefpodoxim	sinusitis	+	toxin +	1998	Veillard et al., 1998
27	2,5	m	Knee	amoxicillin, cotrimoxazol	otitis media	n.d.	toxin +	1997	Cron and Gordon, 1997
28	3	m	Hip	cefixim	otitis media, rhinitis	-	toxin +	1997	Finger and Neubauer, 1997
29	26	m	oligoarthralgia	erythromycin, amoxicillin/ clavulanic acid cotrimoxazol, oxacillin	sinusitis	+	toxin A +	1995	Keating and Vyas, 1995
30	66	m	Polyarthralgia	penicillin	tooth abscess	+	culture + toxin +	1994	Boice, 1994
31	61	f	Polyarthralgia	ampicillin, gentamicin, metronidazol	sigmadiverticulitis	-	toxin +	1993	Putterman and Rubinow, 1999
32	39	m	Oligoarthralgia	ceftriaxon, cotrimoxazol, bacitracin/ neomycin	postoperative prophylaxis after hernia surgery	n.d.	toxin A/B +	1993	Sensini et al., 1993
33	48	m	Polyarthralgia	oxacillin	infected insect bite	+	toxin +	1992	Cope et al., 1992
34	56	f	Oligoarthralgia	cephalexin	urinary tract infection	+	culture + toxin +	1990	Hayward et al., 1990
35	23	f	Oligoarthralgia	clindamycin	infection of the upper respiratory tract	+	culture +	1989	Hannonen et al., 1989
36	36	f	Oligoarthralgia	clindamycin	sialadenitis	-	culture +	1989	Hannonen et al., 1989
37	45	m	Hand	penicillin, clindamycin	tonsillitis	+	culture +	1989	Hannonen et al., 1989
38	29	f	Polyarthralgia	clindamycin, cefoxitin	bacteremia after Cesarean section	+	toxin +	1989	Mermel and Osborn, 1989
39	44	m	Oligoarthralgia	cloxacillin	cellulitis after insect bite	+	culture + toxin +	1988	Atkinson and McLeod, 1988
40	22	m	Polyarthralgia	penicillin	tooth extraction	-	culture + toxin +	1988	Atkinson and McLeod, 1988
41	28	f	Polyarthralgia	none	none	-	culture – toxin +	1987	Paty and Nichols, 1987
42	61	m	Oligoarthralgia	cephalexin	perioperative prophylaxis	+	toxin +	1984	Lofgren et al., 1984
43	27	f	Polyarthralgia	amoxicillin	fever after surgery of the uterus	-	culture + toxin +	1982	Abbott and Caughey, 1982
44	37	f	Polyarthralgia	clindamycin	combustion	+	culture + toxin +	1982	McCluskey et al., 1982
45	45	f	Oligoarthralgia	clindamycin	infection of the finger	-	culture + toxin +	1981	Bolton et al., 1981
46	53	f	Polyarthralgia	lincomycin, gentamicin	anorectal surgery	n.d.	culture + toxin +	1980	Fairweather et al., 1980

*m, male, f, female, n.d., not done, ? = no information available.*

In the 46 patients described in Table 1, *C. difficile* was identified as a potential cause of reactive arthritis by culture (*n* = 21; 45.6%), toxin detection (*n* = 40; 87.0%) or a positive glutamate dehydrogenase (GDH) test from stool specimens (*n* = 2; 4.3%). However, ribotyping of the bacterial isolates was only carried out in two of the 46 patients described so far. The mean age of the affected patients was 37.1 years with an increased occurrence in the age group 21-30 years. There was no clear association of reactive arthritis with the sex of the patients (25 male vs. 21 female). In contrast to our patient, 72.5% of those affected had oligo- or polyarthritis (*n* = 29/40). As in our patient, HLA-B27 was detected in 59.0% of the patients tested (23/39). Like our patient, 89.1% (41/46) of the patients described in Table 1 fulfilled all of the criteria established by Putterman and Rubinow (26) for the diagnosis of CDARA (Table 2). Only five of the affected patients reported no pre-existing antibiosis in their anamnesis.

**TABLE 2:** Criteria for the diagnosis of *C. difficile*-associated reactive arthritis (mod. Putterman and Rubinow, 1999).

Presence of sterile inflammatory arthritis, together with or followed by:
Diarrhea and/or colitis
Previous use of antibiotics
Proof of *C. difficile* or its toxin(s) in stool samples
Absence of alternative explanation/cause for arthritis and diarrhea

The vast majority of patients (*n* = 31/41; 75.6%) took beta-lactam antibiotics in connection with reactive arthritis. Amoxicillin and ampicillin - possibly combined with a beta-lactamase inhibitor - were clearly in the foreground with 29.3% (12/41) of the patients, followed by cephalosporins with 26.8% (11/41) of the patients. Eight of the patients with antibiotics (19.5%) had taken clindamycin.

In contrast to his female partner, who was also infected with the *C. difficile* ribotype 027 and who did not develop reactive arthritis, HLA-B27 was detectable in our patient. He reported having had reactive arthritis after enteric *Salmonella* infection more than 30 years ago. Although determination of HLA-B27 was not done at that time, an association between this phenotypic marker and reactive arthritis is well known making it likely that the patient’s tendency to develop reactive arthritis after infection with defined intestinal pathogens (e.g., *Salmonella enterica* and *C. difficile*) is at least partially linked to HLA-B27 (Bentaleb et al., 2020). Even if this evidence attaches a role to HLA-B27 in the development of post-infectious reactive arthritis, only 59% of the described patients were positive for this biomarker (Table 1), making its precise involvement in pathogenesis still debatable (Bentaleb et al., 2020).

We therefore looked for other possible factors that predispose to the development of reactive arthritis after CDI or that may be suitable as prognostic biomarkers for the development of reactive arthritis after CDI in the future. Toxigenic CDI induces the production of interleukin-23, which - together with IL-17 - plays an important role in the development of inflammatory arthritis. In the context of this IL-23/IL-17 axis, B cells are activated for the production of autoantibodies (Lubberts, 2015). For this reason, we wanted to clarify whether the humoral immunity of the patient and his partner differ. In fact, a significantly stronger humoral immune response to the immunodominant *C. difficile* protein SlpA was detectable in the patient’s serum compared to his female partner. These data are not intended as an explanation for pathogenesis of reactive arthritis but should rather give possible other explanations for the immunological difference between the patient and his female partner. This finding is also consistent with the persistence of IgA antibodies in reactive arthritis after infections with a whole range of other pathogens (Mäki-Ikola et al., 1994). It still remains to be clarified what role these antibodies play in the pathogenesis of CDARA and whether they are suitable as prognostic biomarkers for the development of reactive arthritis after CDI.

## Conclusion

This case shows that CDI should in principle be considered as a rare cause of reactive arthritis. The identification or further development of corresponding biomarkers would be desirable, also to improve the state of knowledge on pathogenesis.

## Data Availability Statement

The original contributions presented in the study are included in the article/supplementary material, further inquiries can be directed to the corresponding author/s.

## Ethics Statement

Ethical review and approval was not required for the study on human participants in accordance with the local legislation and institutional requirements. The patients/participants provided their written informed consent to participate in this study.

## Author Contributions

UG, HK, and OZ had the initial idea to perform this study. OZ, WB, and BP-K collected the samples and performed the laboratory analyses. PP did the clinical investigations. UG wrote the manuscript that was read and approved by all authors.

## Conflict of Interest

The authors declare that the research was conducted in the absence of any commercial or financial relationships that could be construed as a potential conflict of interest.

## Publisher’s Note

All claims expressed in this article are solely those of the authors and do not necessarily represent those of their affiliated organizations, or those of the publisher, the editors and the reviewers. Any product that may be evaluated in this article, or claim that may be made by its manufacturer, is not guaranteed or endorsed by the publisher.
